# Experiences and ethical issues during shared decision-making in healthcare at rural health facilities in Uganda: An exploratory qualitative study

**DOI:** 10.21203/rs.3.rs-4930541/v1

**Published:** 2024-09-16

**Authors:** Ranga Solomon Owino, Nelson K. Sewankambo, Olivia Kituuka, Paul Kutyabami

**Affiliations:** 1 Makerere University Lung Institute, Kampala, Uganda.; 2 School of Medicine, Makerere University, Kampala, Uganda; 3 School of Health Sciences, Makerere University, Kampala Uganda.

**Keywords:** Shared decision-making, healthcare, ethical issues, patient, autonomy, healthcare provider

## Abstract

**Background:**

Shared decision-making in healthcare is a collaborative process where patients are supported to make informed decisions according to their preferences. Healthcare decisions affect patients’ lives which necessitates patients to participate in decisions concerning their health. This study explored experiences and ethical issues related to shared decision-making in a rural healthcare setting.

**Methods:**

An exploratory qualitative study was conducted at Budumba Health Centre III and Butaleja Health Centre III in rural Eastern Uganda. In this study, 23 in-depth interviews were conducted among 12 healthcare providers and 11 patients. Data was analyzed thematically using NVivo-12 plus software.

**Results:**

Four themes emerged which included: experiences, challenges, strategies for improvement, and ethical issues. Patients at both facilities expressed the need to be involved in decision-making processes. However, many stressed that they are not engaged in decision-making about their health. Many healthcare providers noted that shared decision-m aking could improve patient prognosis but are faced with challenges related to low male involvement and the influence of cultural and religious practices, including myths and patriarchal attitudes which impact effective patient engagement.

Ethical issues included concerns about informed consent, privacy and confidentiality, deception, and harm. This study highlighted the need for better sensitization of patients and comprehensive training for HCPs to minimize and resolve ethical issues that emerge during shared decision-making processes.

**Conclusions:**

There is a need for targeted interventions to enhance decision-making processes in rural healthcare including but not limited to developing shared decision-making manual and continuous training of healthcare providers to ethically engage patients. Further research is needed to explore larger facilities with a bigger scope including patients under 18 years of age and and their surrogates.

## Background

Shared decision-making (SDM) is considered a key ethical component in patient-centered care. It has the potential to strengthen medical decision-making and patient-healthcare provider relationships and improve quality, and safety in healthcare for better health outcomes (1,2). It is important to note that many healthcare decisions affect patients’ lives. For better patient satisfaction and treatment compliance, patients ought to participate in healthcare decisions against a backdrop of their rights and unique clinical situations (3).

Patient involvement in SDM emanates from the ethical principle of autonomy. The principle stipulates that individuals with sound minds and the capacity to make rational decisions ought to be involved in decision-making (1). Further, the Kantian theory of rights and duties asserts that “individuals should be treated as ends in themselves and not as mere means to an end”. This implies giving patients the right to make autonomous decisions (4).

For better health outcomes, healthcare providers (HCPs) must inform their patients about the available care options and engage them in SDM to provide appropriate and cost-effective care, lower the risk of healthcare complications, and ultimately lead to patient satisfaction (5,6).

Globally, patient involvement in healthcare decision-making has become a necessity where HCPs collaboratively help patients reach evidence-informed and culture-value-congruent care decisions. Several studies point out that SDM is associated with improved treatment outcomes, enhanced quality of life, and treatment compliance (5,7,8). However, SDM remains a key challenge in the health systems of developing countries. (9).

In Africa, SDM varies across countries and is poorly implemented and the development of supporting tools has not been a priority in many countries (10). In recent years SDM has become essential for the strengthening of healthcare systems and healthcare service delivery at national and sub-national levels. There is, however, no evidence of a growing interest in measuring SDM experiences and finding solutions to improve the SDM processes and address ethical issues that may surround SDM. (11)

In Uganda, despite having the patient’s charter that provides for the patient’s right to make informed decisions about their healthcare, there’s low patient awareness about these rights. This is coupled with a low HCP-to-patient ratio, which makes the feasibility of SDM during clinical consultation time challenging (11). Therefore, there is a need to enable ethical SDM at rural healthcare facilities and patient sensitization to engage in healthcare decision-making processes for better healthcare outcomes. This study aimed to examine the ethical issues and experiences related to SDM in rural healthcare in Uganda.

## Methods

### Study design

This study employed an exploratory qualitative approach to elicit the experiences, and ethical issues surronding SDM in rural healthcare context.

### Study sites

The study sites were Budumba Health Centre III and Butaleja Health Centre III. Both are rural public health facilities located in Butaleja District in Eastern Uganda. These facilities are designed to offer services such as outpatient care, maternity services, inpatient services, laboratory services, health education, and referral to higher facilities like health center IV and hospitals. The selected facilities are located 225 kilometers and 242 kilometers respectively away from Kampala. The selected facilities serve populations with limited access to comprehensive healthcare services and are often constrained by inadequate medical supplies, low patient-to-HCP ratio, and low awareness of patients’ rights.

In such rural contexts, SDM processes are complicated due to factors such as low health literacy, cultural beliefs, and economic constraints, that limit patients’ ability to engage effectively in SDM processes. Therefore, suitable sites to explore patients’ and HCPs’ experiences and ethical issues relating to SDM for developing strategies to enhance SDM practices in rural resource-constrained settings.

### Sample size

In this study, 23 participants were recruited. The sample size was determined based on the principle of data saturation. This was considered a point in the data collection process at which no new ideas were emerging from additional interviews (12).

### Sampling procedure

A purposive sampling technique was used to recruit participants. The participants were selected from clinical officers, nurses, midwives, out-patients, and in-patients.

### Data collection tool

The In-depth interview (IDI) guides used in this study were developed for this research and was not adapted from any pre-existing tools. They were developed based on the unique specific aims of the study, and guided by scholarly views (13,14). The questions asked in the interview guide were on participant perceptions about being involved in SDM, experiences, ethical issues they have encountered, challenges encountered during SDM, and strategies to better SDM in their setting.

To ensure validity and reliability, expert opinions were sought for a deeper review of the IDI guides, and to check the objectivity and clarity of the questions. Two individuals who were not involved in the study were purposively selected for their expert opinions given their knowledge of bioethics, research experience, and their availability to be contacted for their input. The data collection guides were pretested on four participants (02 HCPs and 02 Patients) at Busabi Health Centre III before actual data collection to ensure that the interview guides were relevant to the research questions in context.

### Data collection procedure

Data were collected using face-to-face IDI. Note-taking was done by a trained research assistant (RA) who was a male student of Master of Health Science in Bioethics in his final year and a pharmacist by profession. The RA had a valid certificate of Good Clinical Practice and one year of experience in qualitative data collection in a healthcare setting. Interviews were conducted in a quiet, private, and convenient room after obtaining written informed consent from each participant.

Confidentiality was maintained by assigning each participant a unique study code. All hard copy study-related materials were kept under lock and key and only accessible to the study team. Data stored in soft copy were secured on a password-protected computer for confidentiality purposes.

Data collection tools were translated into *Lunyole* which is the predominant language in the study area. Eight patient interviews were conducted in the *“Lunyole”*. Data collected in lunyole was then transcribed into English. Interviews were audio recorded with the study participants’ consent to do so.

### Data analysis

Audio-recorded data from 23 IDIs were transcribed verbatim. After transcription, the transcripts were independently reviewed by two reviewers (BE and RSO) and then discussed as a group to identify and categorize the codes under themes which included; experiences during SDM, practices and influence of culture in SDM, challenges in SDM, strategies for better SDM, understanding of SDM, and ethical issues during SDM.

The themes were developed inductively based on specific observations. These themes guided the development of initial codes that were exported to NVivo Version 12 plus for coding using a coding framework that was iteratively revised. The COREQ checklist (Table 1) was applied to ensure adherence to criteria for qualitative research (15).

### Ethical considerations

Ethical approval was obtained from the Makerere University School of Biomedical Sciences Research and Ethics Committee, Ref No: SBS-2022–267. Written informed consent to participate in the study was sought from each participant. To ensure anonymity and confidentiality, participant codes were used on data collection materials.

## Results

A total of 23 IDIs were conducted. 12 of the participants were HCPs, seven were female and the mean age of HCPs was 35 years (range 28–52 years). Of the 12 HCPs, four were clinical officers, five were nurses, two were midwives and only one was a counselor. Most HCPs (10/12) had experience in healthcare of up to 10 years. 11 of the participants were patients, six were females and the mean age of patients was 32 years (range 18–53 years). Nine were married, two were single, and only one was widowed. Most of the patients were peasant farmers (8/12).

Four themes emerged from the data: experiences during SDM, challenges in SDM, strategies for better SDM, strategies to improve SDM, and ethical issues.

### Experiences during SDM

#### Patient experiences

Many patients reported that they are hardly involved in decisions about their healthcare. They pointed out that some HCPs don’t inform them about the available options to make informed decisions. However, many patients thought that it would be prudent for HCPs to always engage them in a collaborative approach given that medical decisions at times have devastating health and financial implications to the patient.

“…Yes, I feel it is important for the health worker to involve me in decisions about my health. Because it helps me make my own choices. After explaining to me the treatment, e.g., family planning then I can make my own decision from an informed point”.

(BUT PT 004).

Despite many patients stating that they need to be engaged in SDM, only a few patients reported being actively involved by HCPs in decisions regarding their health. To stress this further, patients noted that sometimes they are not even informed of the treatment procedures and available options if any.

“When I came to this facility, the health worker did not involve me in any decisions. And when the decision is made, I have nothing to change; whatever they decide is what they do”.

(BUD PT 006).

#### HCP experiences

Some HCPs indicated that maximum cooperation in decision-making facilitates adherence and a better prognosis. They noted that when a patient understands and agrees on a treatment option, treatment may go well. Many HCPs highlighted that when patients fail to understand available care options, it conflicts with their decision-making process. One HCP noted that in some cases it is hard to provide options to patients because at times there is only one affordable or available option.

“…You will find it hard to involve the patient if this person is not accepting what you want, yet you need to help him or her. So, then you need much time explaining to one patient yet you have other patients waiting”.

(BUT HCP 004).

Most HCPs noted that SDM is very important in healthcare. They highlighted that SDM can help in attaining a better treatment outcome, including an improvement in diagnosis. It can also help HCPs understand the financial situation of a patient; the culture, views, or the patient’s stand; and the patient can be advised or empowered to make individual decisions. One HCP added that SDM can help to analyze the quality of services and help HCPs, policymakers, and other stakeholders bridge the gaps in clients’ satisfaction with healthcare services.

“…They need to be involved in shared decision making not only because of the financial implications but, they have to be involved to achieve the most appropriate healthcare decision considering all factors that come into play”.

(BUD HCP 001).

#### Health-seeking behavior and men’s Involvement in healthcare

Several HCPs pointed out that women’s health-seeking behavior differs from that of men. They noted that despite the power that men have in society in decision-making, their involvement in healthcare is still very low. This poses a challenge in situations where female patients might need the consent of the husband for services such as family planning. It is important to note that, some married female patients emphasized the relevance of their husband’s involvement and support in arriving at healthcare decisions.

“Men’s involvement in health issues is very, very low. By the time a man is brought into the facility to seek health interventions, it is usually very late or when he is critically ill. So, men are rare in this thing of seeking healthcare yet they control the economic muscle. So, you find that it is the men that influence decision-making”.

(BUD HCP 001)

#### Influence of culture and religious practices

Many HCPs stated that cultural beliefs affect their daily duties. They noted that in most cases, some people do not believe in Western medicine or treatments. Instead, they tend to believe in witchcraft, which then contributes to some cases of treatment refusal. Similarly, one HCP stated that some religious beliefs influence their daily activities as some contradict required treatments, such as blood transfusions, that are prohibited in such religions;

“And also, in some religions, a patient might need blood to be transfused, but then is like in our faith, we don’t allow blood to be transfused. So, it is not always easy to engage such patients, but keep encouraging them”.

(BUD HCP 006).

### Challenges during SDM

#### Patients’ and HCPs’ mistreatment

Some patients reported that they are not well treated at healthcare facilities because some HCPs are rude, don’t listen to patients, or may attempt to engage in love affairs with patients. On the other hand, some HCPs revealed that some patients are abusive and arrogant and they don’t listen to what HCPs tell them.

“Some health workers at times are not all that hospitable and social. Someone comes to discuss with you about his or her illness. And instead of the health worker listening to the patient’s suggestions, they are rude, tough, and at times quarrelsome. So, as a patient, you fail to express your view freely to them”.

(BUD PT 003)

“I think it’s sometimes arrogance. Like I told you. Some patients come to the hospital while stressed. So when you engage them they just abuse you”.

(BUD HCP 005).

#### Inadequate time and patient willingness to engage in SDM

Some HCPs highlighted that engaging patients in SDM is challenged by limited time, making it hard to engage patients in intensive discussion and provide enough information to make decisions on various issues. Also, they added that sometimes patients refuse or fear engaging in SDM.

“We always have a problem with time to engage each patient because in most cases, some patients are in a hurry. So, you then have limited time to engage them into shared decision making”.

(BUT HCP 003).

#### Limited resources

Many HCPs noted that there are limited resources including a shortage of medical equipment, drugs, and human resources leading to a huge workload. This contributes to less time spent on discussion of different issues with patients. In addition, the limited workspace is not conducive to patients’ privacy.

“Yes, the nurse-patient ratio is not okay with us, and we also have limited space for privacy to ensure patient confidentiality”.

(BUD HCP 003).

### Strategies for better-shared decision-making

#### Good relationship between HCPs and patients

Many patients suggested that a good relationship between HCPs and patients is one of the best ways to improve SDM and strengthen this relationship. Also, building rapport was considered vital to enabling socialization and receiving various views from patients involved in decision-making. Patients suggested that HCPs should be free and open with patients, as this facilitates SDM.

“All in all, there needs to be a good relationship between the patient and the health worker for productive engagement to take place”.

(BUT PT 006).

#### Government involvement and support

HCPs noted that there are challenges that are beyond their means and emphasized the need for government involvement to address the challenges. They pointed out that the challenges include ethical and infrastructural issues. So, there is a need for government support through the Ministry of Health to aid in addressing some of the challenges hindering the ethical engagement of patients at rural facilities.

“We need continuous support because sometimes we get ethical challenges, we feel we cannot handle as we try to engage patients in decision-making”.

(BUT HCP 003).

#### Patient sensitization and counseling

Many patients and HCPs highlighted the need for proper sensitization programs through patient education to create awareness about patient rights and the benefits of SDM. They noted that this could be attained through public media like radio and television, as well as other appropriate communication channels. Others suggested that there is a need to have well-trained, counselors at every healthcare facility because most HCPs may not have the required expertise to adequately address the ethical issues that emerge.

“Patient sensitization is also needed because some patients reach the hospital but are ignorant of their rights. So, there’s a need to raise community awareness about the rights that I, as a patient, have.”

(BUD PT 003).

“Now, I say that we need to be well-equipped in terms of personnel. Now, in this unit here we don’t have professional counselors to help resolve some technical issues. Because there are areas where you may find that they are too technical to handle. Yeah, especially things to do with ethics…”.

(BUD HCP 004)

### Ethical issues during SDM

#### Informed consent

Most HCPs highlighted that patients should provide informed consent before administering certain treatments; hence, they should decide for themselves, and HCPs must respect their decisions. They noted that it is essential to inform patients that they are supposed to consent in all cases rather than in cases that need extremely urgent action. However, to ensure that this is effected, it may be necessary to demonstrate to the patients the available treatment alternatives. One of the HCPs indicated that sometimes she brings actual materials for demonstrations to enable patients to comprehend and make their own decisions.

“I bring the real things that we are going to use. Like, let me say, family planning, you get all those things, then you show them, like a condom, or the implant, So, we present to them the available options and they make their own decision”.

(BUD HCP 002).

On the other hand, some HCPs noted that sometimes they decide for patients upon their request. In some circumstances, HCPs force patients to accept a certain kind of treatment, even when the former knows it is inappropriate to do so.

“There are some situations where you have to apply some force, but the forcing should also be in line with the medical practice and somewhere within the legal terms”.

(BUD HCP 004).

#### Delivering care or treatment options

Most HCPs revealed that they provide treatment options to give patients a chance to choose what they want. Before offering the options, they typically educate patients and discuss with them about potential side effects of available treatments. However, some HCPs noted that they don’t provide treatment options to patients because of the severity of a particular illness the HCP’s preference for one treatment over another, or because there is only one treatment option available. An experienced senior clinical officer and health counselor explained;

“But when it comes to treatment options, what is always available is what we give; we don’t always have an alternative for the patient to make a choice”.

(BUT HCP 001).

“…So sometimes it comes back to the judgment of the health worker in most cases depending on the severity of the illness. That’s why it is hard to present available options to these patients…. Incidentally, for family planning most commodities are available, and in plenty so that is the contradiction. So, that you can offer them the options. And they choose from those available options because usually the options are in a wide range and they are usually in stock”

(BUT HCP 004).

The information above from a senior clinical officer that HCPs may not offer patients treatment options and instead choose for them was collaborated by some patients as a patient aged 19, alluded that;

“The health workers don’t share the available options before treating a patient. When you’re a patient, the health workers just decide for you because they know what is best for the patient”.

(BUT PT 003).

#### Concerns about privacy and confidentiality

Many participants both patients and HCPs highlighted that privacy and confidentiality are not always observed during treatment. This was mainly attributed to limited working space at the health facility; for example, there are no separate dedicated sections for male and female patients, so patients share the same ward. Also, HCPs attending to many patients simultaneously violate patients’ privacy. On the other hand, participants noted that maintaining privacy and confidentiality is challenged by the attendant’s or caretakers’ demand to know their patients’ results or treatment progress. However, some HCPs stressed that they usually try not to keep patient information confidential.

“Sometimes, like a nurse here in the maternity ward, you may come when you want to tell her something in private, but then she talks to you when you are like three people, so you get afraid to share with her personal information”.

(BUT PT 001).

#### Sharing of information

HCPs stated that sharing different types of information among HCPs and patients is essential. They suggested that sharing information can facilitate agreement because patients will be well-informed and agree with HCPs on different treatment options. Information sharing also empowers patients to be aware of many things, which helps them make good decisions. On the other hand, HCPs noted that they share information among themselves when there is a sensitive case before sharing it with patients.

“My experience with sharing information and agreeing with the patient is that you get maximum adherence and a good prognosis”.

(BUT HCP 001).

#### Deception and doing harm

Some HCPs reported that sometimes a few patients request them to be deceitful more when such patients want to hide something from their spouses, families, or caretakers. For instance, one HCP in her late twenties shared her experience concerning deception;

“Sometimes a patient can come and tell you. You know what, I’ve been with my husband for this period. But I’ve never conceived. So, I want that when I come together with him, just tell him that I am pregnant, and then after a short period, I will come back, and you will say, I had a miscarriage”.

(BUT HCP 003).

## Discussion

### Experiences during SDM

Our findings suggest that SDM is not well practiced in the rural health centers we studied. In Uganda, there are no national guidelines for SDM in healthcare and neither were they present at each of the study sites. This hampers the ideal practice of SDM to improve patient outcomes. Only a few patients actively participate in SDM during healthcare. This is due to several reasons for example the low literacy of the patients, their low willingness to participate in SDM, and an unfavorable healthcare environment that does not provide adequate time for discussion and socialization with HCPs.

There are structural and capability barriers to the implementation of SDM which include, poor communication skills, HCPs’ and patients’ attitudes towards SDM, and the communities’ cultural and religious beliefs and practices, which contribute to failure to agree on various treatment options. These findings are similar to those identified in the study by Vedasto et al (2021) conducted in Tanzania on SDM between HCPs and patients at a tertiary hospital in the diabetic clinic, where many participants reported not having participated in SDM.

Patient involvement in SDM has been emphasized as a crucial aspect of contemporary healthcare (16). Our findings suggest that in some cases, HCPs decide for patients, and yet many patients expressed the need to be more actively engaged in SDM. In part, the way HCPs handle patients determines the latter’s involvement in SDM. Patients can be motivated to talk and discuss with HCPs who provide helpful information. This is in line with the self-determination theory which asserts that people are more motivated to act when they believe their actions will have an impact on the outcome. According to this theory, when individuals whose requirements for competence, connection, and autonomy are met, they can become self-determined (17). Therefore, there is a need for HCPs to improve the way they engage patients in SDM by creating socialization.

In a multicultural society, healthcare professionals often have to deal with people from different cultural heritages. This affects how a situation is explained or understood, how a problem is perceived, and what a critical decision-making argument is (18). Cultural and religious practices were reported as important factors influencing SDM. HCPs reported that most patients believe in cultural and religious practices that may contradict the intended formal treatment. When cultural and religious practices contradict Western medical treatment, it becomes quite challenging because both sides may not agree on different treatment options, and hence the HCP’s actions will not benefit the patients.

Additionally, health-seeking behavior was considered an essential factor influencing SDM. HCPs reported that men’s involvement in healthcare services is low, despite being the financial controllers within their families and making most healthcare decisions. There is a need for proper guidance of HCPs on how to engage patients and caretakers to appropriately deliver treatment options.

Other challenges in the SDM process also require corrective action by the health facilities and HCPs. These include overcoming, and avoiding the mistreatment of patients, improving human resources to enable sufficient time for participating in the discussion, and availing more resources such as better infrastructures, and more funding for drug supply to foster the feasibility of treatment options.

On the other hand, reaching an agreement should be attained respectfully, where all views or opinions will be listened to and respected without showing inappropriate behavior towards each other. Studies suggest that good relationships where HCPs socialize with patients lead to a positive patient experience, promote patient satisfaction, and help optimize efforts (19–21). Good relationships between HCPs and patients can be achieved based on good government strategies like proper sensitization of communities at large along with HCP counseling are essential in enforcing SDM (22). To achieve this, media such as radio and television can be used to inform and sensitize the general public as recommended by both patients and HCPs.

The government should act within its means and increase human resources by employing more health counselors and providing basic continuous training to HCPs in communication skills. Government strategies should go hand in hand with the involvement of different stakeholders, and appropriate policy formulation informed by the best affordable methods and alternatives to address the identified challenges.

It is very encouraging that all participants had an appreciation of the potential value of implementing SDM in healthcare services. This suggests that if the existing barriers are addressed, several benefits like attaining better treatment outcomes and improving the quality of healthcare services would be realized. Our findings are similar to the study by Ambigapathy et al, conducted on patient involvement in decision-making in a Malaysian primary care clinic where more than half of the patients preferred SDM (23).

To facilitate SDM, both sides should have responsibilities and abide by them. HCPs should communicate appropriate information, as well as listen to the patients’ views or opinions and provide a chance for the patient to ask questions where necessary. However, given that several HCPs stated that they were unaware of any available shared decision model. It would be prudent that they adopt and put to use the pragmatic three-step guide on SDM (24). To promote patient-centered care, many healthcare experts and organizations encourage HCPs to move towards an SDM model (25). This approach could be guided by the step SDM communication model proposed by Elwyn et al. This ideal SDM model in clinical practice includes a choice talk, an option talk, and a decision talk (26).

### Ethical issues surrounding SDM in rural healthcare

The SDM process is an ethical imperative in the HCP-patient relationship and should be guided by the ethical principles of autonomy, beneficence, non-maleficence, and justice (27). The study findings suggest that in most cases, patients do not consent to their treatment. In this case, the HCPs tend to decide for patients. However, efforts should be made to reach a mutually agreeable decision for the patient and HCPs (28). Thus, a balance of autonomy and beneficence is achieved but in certain circumstances where mutually agreeable SDM may be impossible. In this case, the HCP should inform the patient of alternatives, if appropriate, such as transfer to another HCP or facility (27).

During clinical encounters, patients share sensitive information with HCPs. The HCPs have an ethical, legal, and professional duty to protect patient’s privacy and confidentiality (29). Sections 1 and 2 of the Ministry of Health (Uganda), Patients’ Charter 2009 emphasize the rights and responsibilities of patients, which aim to bring about awareness of patient’s rights and responsibilities that have been lacking among the population of Uganda (30).

The HCPs ought not to deceive or do harm to their patients in line with the principle of non-maleficence. Failure to observe normative ethical principles in SDM undermines medical professionalism and patient-centered deontological theory that is premised on peoples’ rights as opposed to mere duty. HCPs should therefore perform acts that conform to a moral norm as the guiding principle (31). There is also a need to ensure that SDM is conducted in a favorable environment to ensure ethicality regarding its process and final decision.

## Strengths and limitations

The strength of this study is that it was conducted in two rural lower-level health facilities (Health Center III) and this is where most people in Uganda seek their primary healthcare. The findings are very relevant in this context and serve to inform how the quality of healthcare can be improved at this level. This study’s limitation is that it included only adult patients in scope (>18 years of age) leaving out those below the age of 18 and their surrogates. Some of the older minors may be capable of participating in SDM and for others, their surrogates should be provided with the opportunity to participate in SDM.

Whereas SDM is still relevant when providing healthcare to minors, data is lacking on the ethical issues surrounding involving minors in SDM in Uganda. To bridge this knowledge gap, future studies centered on minors may be conducted.

## Conclusions

This study underscores the need for enhanced patient involvement in SDM at rural health facilities. Patients overwhelmingly express a desire to participate in decisions about their health, yet many reported inadequate engagement in this process. HCPs noted the benefits of SDM for improving patient outcomes however, emphasized that they encounter significant challenges, including low male involvement and the impact of cultural and religious beliefs.

Ethical concerns to do with informed consent, privacy and confidentiality, deception, and harm identified highlight the necessity for increased patient sensitization, the development of an SDM manual, and comprehensive training for healthcare providers to advance effective and ethical SDM practices in rural healthcare settings.

## Data Availability

All data analyzed for this study will be freely accessible from the corresponding author at reasonable request.

## What is known about this research topic:

It is known that shared decision-making has the potential to improve patient care. However, several ethical issues related to informed consent and autonomy, power dynamics, justice, confidentiality, and privacy emerge during shared decision-making processes.

## What does this study add:

This study provides empirical data on the specific ethical challenges in rural healthcare settings. It highlights the gaps in current practices and underscores the need for continuous training of healthcare providers and patient sensitization to improve shared decision-making processes in rural Uganda.
